# Effect of Seed Coating and PEG-Induced Drought on the Germination Capacity of Five Clover Crops

**DOI:** 10.3390/plants10040724

**Published:** 2021-04-08

**Authors:** Antonín Kintl, Igor Huňady, Tomáš Vymyslický, Vladěna Ondrisková, Tereza Hammerschmiedt, Martin Brtnický, Jakub Elbl

**Affiliations:** 1 Agricultural Research, Ltd., 66441 Troubsko, Czech Republic; kintl@vupt.cz (A.K.); hunady@vupt.cz (I.H.); vymyslicky@vupt.cz (T.V.); ondriskova@vupt.cz (V.O.); 2 Department of Agrochemistry, Soil Science, Microbiology and Plant Nutrition, Faculty of AgriSciences, Mendel University in Brno, 61300 Brno, Czech Republic; tereza.hammerschmiedt@mendelu.cz (T.H.); Martin.Brtnicky@seznam.cz (M.B.); 3 Faculty of Chemistry, Institute of Chemistry and Technology of Environmental Protection, Brno University of Technology, 62100 Brno, Czech Republic; 4 Department of Geology and Soil Science, Faculty of Forestry and Wood Technology, Mendel University in Brno, 61300 Brno, Czech Republic; 5 Department of Agrosystems and Bioclimatology, Faculty of AgriSciences, Mendel University in Brno, 61300 Brno, Czech Republic

**Keywords:** legumes, drought tolerance, polyethylenglycol, hardseededness, dead seeds, seeds improvement

## Abstract

The effect of coating the seed of clover crops by water absorbing seed process (WASP) technology pelletization on its germination capacity was studied in conditions of diverse drought intensities simulated by different concentrations of polyethylenglycol (PEG) 8000 solution. Drought resistance was monitored in the seed of five fodder clover species: *Anthyllis vulneraria* L., *Medicago lupulina* L., *Trifolium repens* L., *Melilotus albus* Medik. and *Onobrychis viciifolia* Scop. In the seed of given plant species, germination capacity was determined along with the share of dead and hard seeds. Although the coating significantly (*p* < 0.05) affected the drought resistance of seeds, the germination capacity increased only in conditions of milder drought (simulation with PEG: 0.1–0.3 mol). With the increasing intensity of drought induced by higher PEG concentrations (0.4–0.7 mol) the number of germinable seeds demonstrably decreased and the number of dead seeds increased in the coated seed as compared with the uncoated seed. The coated seed can be appropriate for use in *M. lupulina*, *M. albus* and *T. repens*, while the uncoated seed can be used in *A. vulneraria* and *O. viciifolia*.

## 1. Introduction

Seed quality is an important factor upon which the creation of optimum crop stand depends and, hence, the production quality and quantity. Increasing requirements for seed quality have led to the search for new methods for seed improvement. In addition to the production of high-quality seed, the highest possible uniformity of physical characteristics is required (shape, size, weight) as well as the highest possible seed value (purity, germination capacity) [1]. Apart from the process of after-harvest treatment (purification and calibration), very important are also special pre-sowing treatments focused on enhanced seed uniformity, uniform germination and emergence, or on facilitating the subsequent seed handling, which would allow a better distribution of seeds at sowing so that a uniform and precisely established, evenly developing stand is achieved [2]. All seed treatments leading to a precisely established stand can support the use of legumes within a mixed culture system [3,4].

There are three main groups of pre-sowing treatments: moistening treatments (pre-germination) of seeds, biological treatments of seeds (use of fungi and bacteria for the control of soil pathogens and seed-borne pathogens), and coating of seeds (so-called pelletization). Pelletization is the coating of seed with a layer of inert material that alters the original seed shape and size, thus increasing its weight, facilitating the seed handling and sowing. In order to improve the performance of seeds, inoculants, bio-stimulating agents, fungicides and fertilizers are added to the pellet [5].

The coating of seed makes it also possible to eliminate the impact of drought or salinization by improving the availability of water for sown seeds during the period of germination and emergence [6]. Water deficit is one of the important factors preventing seed germination in field conditions [7]. Drought in the period of germination can significantly affect the quality of emerging vegetation and, subsequently, the planned yield [8]. Drought-induced stress affects germination through the constrained water absorption by seeds by acting on the movement and transfer of nutrient reserves and on the synthesis of proteins in germs [7,9,10] This is why hydro-absorbents based on polymers are added to coat layers, too, which bind water and release it gradually, thus supplying the seeds over a long term, even in conditions of uneven water supply or drought. Gel developed from hydro-absorbents can protect the root system of plants from damage by drought. Depending on a specific polymer type, these substances are able to bind up to 250 mL of water per 1 g of their weight. As mentioned by [11], hydro-absorbents play an important role not only in the germination of plants, but they positively affect also the transpiration of plants thanks to the increased availability of water, providing water to nitrogen fixing micro-organisms and affecting the production of both green and dry plant biomass.

In gardening and farming, the coating of seeds is commonly used in vegetables, sugar beet and flowers [12]. No study dealing with the effect of seed coating on the germination capacity in conditions of drought-induced stress is known to have been conducted for clover crops so far. Forage legumes represent an important plant family (Fabaceae) with a potential to provide a sustainable solution for the availability of food products and feeds with a high content of proteins [2]. A successful use of these crops in semiarid regions, i.e., also in the conditions of central Europe, depends on the fast and uniform germination of seeds, which is strictly associated with the capacity of seed germination in case of lacking water [13,14,15].

One of the possibilities for adaptation to the impact of drought and weather extremes, aiming to maintain quantity and quality of agricultural production, is the selection of species and varieties naturally capable of resistance to these stressors. The studied clover species do not belong in the group of highly domesticated crops for human nutrition, e.g., peas and lentils [16,17,18] in which some features such as seed dormancy and cracking of pods in legumes were lost during the process of domestication [19]. This is why a certain share of hard seeds still occurs in these crops.

Hardseededness of clover crops is in fact a physically conditioned type of dormancy the mechanism of which is based on impermeability of testa (seed coat) to water, which induces swelling and germination. In the context of evolution adaptation, it is a strategy of plants for stopping or limiting physiological processes leading to germination in order to reduce the risk of plant death and possible species extinction in the unfavorable environment [20] with the occurrence of drought or low temperatures [21].

In wild plants that remain on the same site for a long period and regenerate stands also by the gradual germination of hard seeds, hardseededness can be considered a favorable characteristic, important for their survival [22]. In a majority of cultivated clover crops, however, even germination and emergence of seeds are required. A high share of hard seeds in the seed stock of clover species obstructs the development of closed and evenly growing stands, hardseededness being considered an undesirable seed stock feature [23].

Drought stress is most often simulated by polyethylenglycol (PEG). The PEG osmotic contains oligomers and polymers with a molar weight of up to 20,000 g/mol and is prepared by ethylenoxide polymerization. PEG causes osmotic stress and can be used as a drought simulator [24], inert osmotic in germination tests [9] and non-penetrating solution [25]. Osmotic stress obstructs seed germination by reducing water absorption [26]. PEG decreases the hydrolysis of nutrient reserves in the seeds and finally the seed germination percent [27]. Methods of testing drought resistance in vitro facilitate progress in understanding the tolerance of plants to drought and help select drought-resistant species and varieties [8]. For testing drought resistance, legume species were selected with the high value added both for their use in animal nutrition and/or biogas production, and for their soil-improving properties: *Anthyllis vulneraria*, *Medicago lupina*, *Trifolium repens*, *Melilotus albus* and *Onobrychis viciifolia,* in which an assumption exists that if their emergence rate can be increased at increased drought intensity, the profitability of their growing and applicability in agriculture will be increased, too [13,14,15,16,17].

The goal of this study was to determine the effect of coating the seed stock of selected clover crops by WASP (water absorbing seed process) technology pelletization on its germination capacity in conditions of diverse drought intensities induced by different concentrations of PEG 8000 solution. At the same time, the effect of coating the seed stock by WASP technology and drought simulated by PEG solutions on the number of dead and hard seeds was assessed. The study can be considered singular as it explores a simultaneous action of two factors on seed stock germination—coating of seeds and simulated drought.

## 2. Results and Discussion

The measured data were evaluated at several levels with different distinctions of experimental factors: Comparison of the proportions of germinated (GS), dead (DS) and hard (HS) seeds in the uncoated (US) and coated (CS) seed stocks (1) with no distinction of individual species and with no drought simulation; (2) with no distinction of individual species and with the drought simulation (PEG treatment at concentrations from 0.0–0.7 mol); (3) with the distinction of individual species and with the drought simulation (PEG treatment at concentrations from 0.0–0.7 mol). Thus, overall results were compared (GS, DS and HS) and then the results achieved in the respective crop species are discussed.

### 2.1. Comparison of the of Germinated, Dead and Hard Seed Percentage in US and CS Treatments without the Distinction of Respective Crop Species (Average of All Species) and without the Simulation of Drought (Control = 0.0 mol PEG)

**Figure 1:**
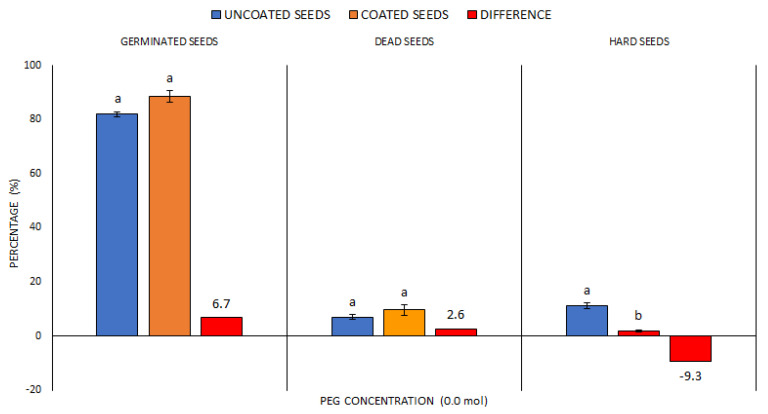
The comparison of germinated, dead and hard seed percentages at control treatment—without drought simulation. Average values for all indicator plants for the US (uncoated seeds; n = 160 ± SE; average of all plant species) and CS (coated seeds; n = 160 ± SE; average of all plant species) treatments with the polyethylenglycol (PEG) concentration = 0.0 mol; different lowercase letters indicate significant difference (ANOVA post-hoc Tukey’s HSD test; *p* < 0.05) for the respective parameters between the US and CS groups.

Average values were compared for all indicator plants whose seeds were used, and all data were divided into two groups—US and CS. The average germination value for all five plant species (Figure 1) in the US group was 81.9%. The average GS percentage value for all five plant species in the CS group was higher—88.5%. However, no significant difference was found. Subsequently, significant differences in the representation of DS were determined. Although the values were different again, they were only partly statistically significant, with the CS group showing a demonstrably lower value than the US group. The two groups reached the same amount in the representation of DS, with no significant differences.

On the one hand, the measured data indicated that the WASP technology could have positively increased the capacity of plant seeds to germinate in the control conditions without the drought simulation. On the other hand, however, regarding the absence of significant differences in GS and DS parameters between the US and CS groups, it is possible to state only the positive influence of seed coating technology on the reduction of HS value because the decrease was greater than 9%.

### 2.2. Comparison of of Germinated, Dead and Hard Seed Percentages in US and CS Treatments without the Distinction of Respective Plant Species (Average of All Species) with the Simulation of Drought (PEG = 0.0–0.7 mol)

**Figure 2:**
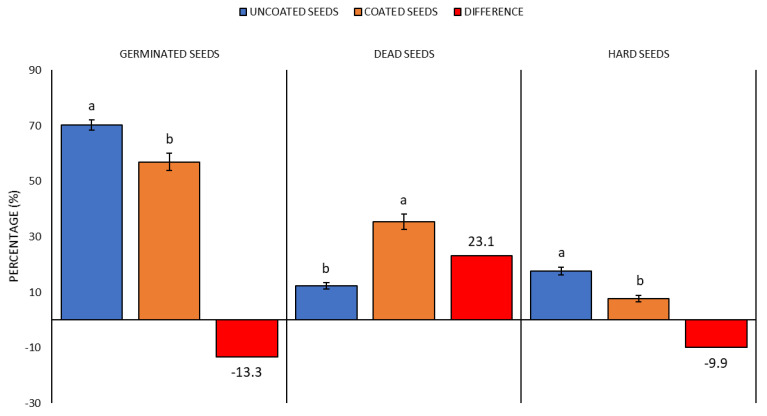
Comparison of germinated, dead and hard seed percentages at PEG treatment—general overview of drought simulation. Average values for all indicator plants for the US (uncoated seeds; n = 160 ± SE; average of all plant species) and CS (coated seeds; n = 160 ± SE; average of all plant species) treatments with the PEG concentration = 0.0 mol; different lowercase letters indicate significant difference (ANOVA post-hoc Tukey’s HSD test; *p* < 0.05) for the respective parameters between the US and CS groups.

With the simulation of drought (PEG concentrations = 0.0–0.7 mol), the overall average representations of GS, DS and HS were affected by the chosen seed coating technology, which was documented by the measured values (Figure 2). The measured values indicated (*p* < 0.05) that the percentage content of GS in the CS treatment decreased by 13%, compared with the US treatment. Apart from this, a significant increase of DS percentage was observed in the US treatment

**Figure 3:**
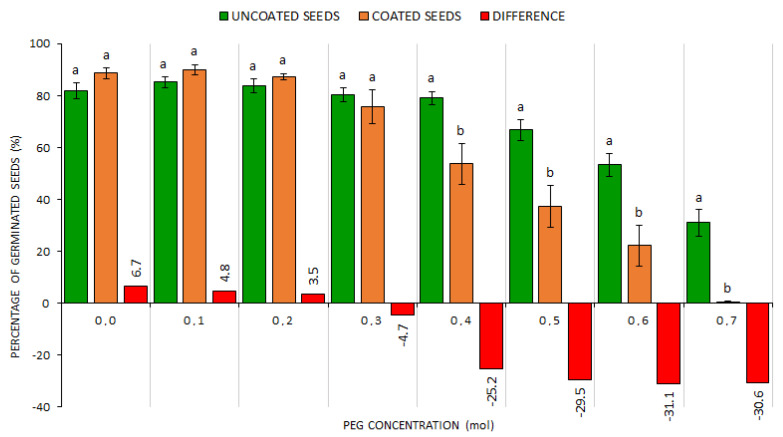
The comparison of germinated seed percentages within US and CS treatments at different drought simulation degrees. Average values for all indicator plants for the US (uncoated seeds; n = 20 ± SE; average of all plant species) and CS (coated seeds; n = 20 ± SE; average of all plant species) treatments with PEG concentrations = 0.0–0.7 mol; different lowercase letters indicate significant difference (ANOVA post-hoc Tukey’s HSD test; *p* < 0.05) for the respective parameters between the US and CS groups.

Taking into account the overall average values of GS, ND and HS percentages at PEG 0.0–0.7 mol, the WASP technology cannot be recommended for increasing resistance to drought. Nevertheless, if the values are compared from the perspective of concentrations of individual PEG solutions (Figure 3), i.e., from 0.0 to 0.7, it is obvious that in certain phases of drought simulation the WASP technology contributed in the CS treatment to the increase of GS value from +6% with PEG 0.0 mol up to +3.5% with PEG 0.2 mol; the germination increases were, however, statistically non-significant. The other differences were already statistically significant and indicated the decreasing germination in the CS treatment as compared with the US treatment for PEG concentrations of 0.4–0.7 mol, with an average difference of 29%, to the detriment of the CS treatment.

**Figure A1:**
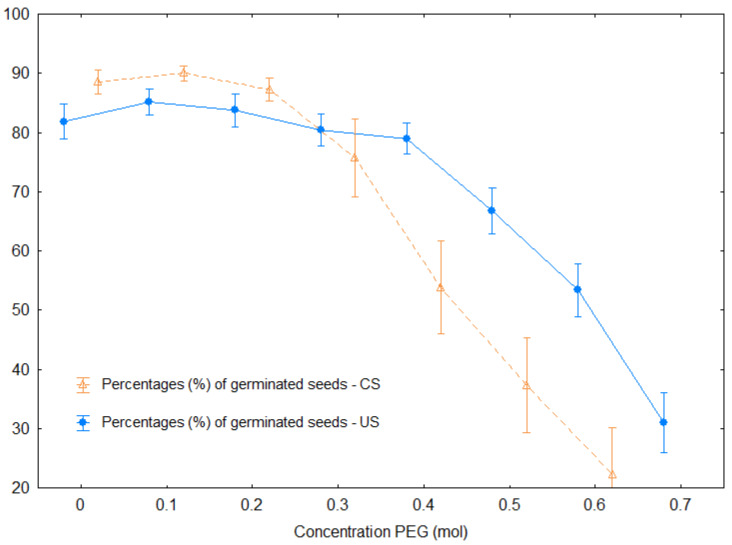
Mean values of seed germination capacity in the US and CS treatments at different PEG concentrations. Average values are shown for all indicator plants for the US (n = 20 ± SE) and CS (n = 20 ± SE) variants at the respective PEG concentrations of 0.0–0.7 mol. US = Uncoated Seeds, CS = Coated Seeds.

In the US treatment, a distinct germination decrease occurred with the PEG concentration of 0.5 mol with the difference in GS percentage values between the concentrations of 0.4 (79.0%) and 0.5 mol (66.8%), which was statistically significant. Differences in germination capacity between the PEG concentrations 0.0–0.4 mol were not significant, which is apparent not only from Figure 3, but particularly from data in Appendix A, Figure A1. The CS variant showed a GS percentage decrease already at the concentration of 0.4 mol, with the difference in germination capacity between the concentrations 0.3 (75.7%) and 0.4 mol (53.8%) being statistically significant. Differences in germination capacity between the PEG concentrations 0.0–0.3 mol were not significant (Appendix A, Figure A1). The PEG concentrations >0.5 mol (wt > 15%), which already simulated severe drought [28,29] in both treatments (US and CS), strongly inhibited germination. A comparison of all clover crop species showed that the average GS percentage values indicated the beneficial influence of seed coating technology only potentially, namely, at lower drought intensities (PEG up to 0.4). From the overall perspective, the coating had no positive influence on the seed resistance to drought (Figure 2 and Appendix A, Table A1). As to the maintenance of germination capacity, the seed drought resistance was significantly decreasing with the increasing drought intensity (Figure 3 and Appendix A, Figure A1).

**Figure 4:**
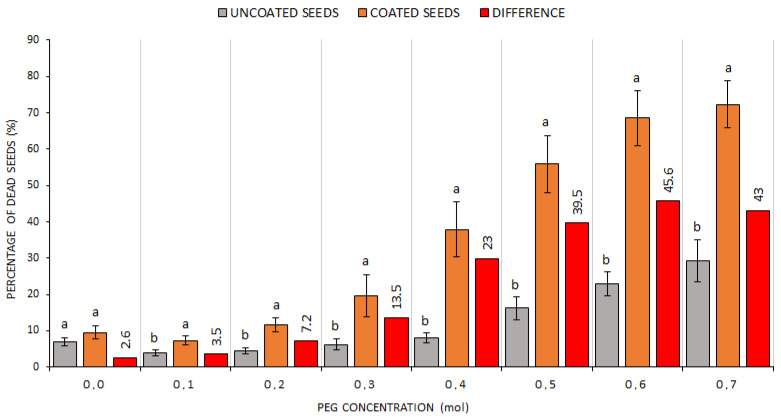
The comparison of dead seed percentages within US and CS treatments at different drought simulation degrees. Average values for all indicator plants for the US (uncoated seeds; n = 20 ± SE; average of all plant species) and CS (coated seeds; n = 20 ± SE; average of all plant species) treatments with PEG concentrations = 0.0–0.7 mol; different lowercase letters indicate significant difference (ANOVA post-hoc Tukey’s HSD test; *p* < 0.05) for the respective parameters between the US and CS groups.

**Figure 5:**
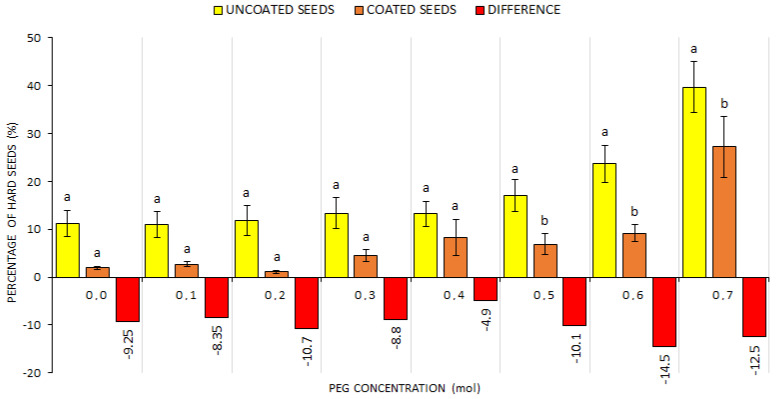
The comparison of hard seed percentages within US and CS treatments at different drought simulation degrees. Average values for all indicator plants for the US (uncoated seeds; n = 20 ± SE; average of all plant species) and CS (coated seeds; n = 20 ± SE; average of all plant species) treatments with PEG concentrations = 0.0–0.7 mol; different lowercase letters indicate significant difference (ANOVA post-hoc Tukey’s HSD test; *p* < 0.05) for the respective parameters between the US and CS groups.

In addition to the GS percentage, the share of dead (Figure 4) and hard (Figure 5) seeds was ascertained in the respective treatments after drought simulation with PEG. It can be considered interesting that there were a significantly higher share of dead seeds in the CS treatment (PEG > 0.1) and a demonstrably higher share of hard seeds in the US treatment at simulated drought (PEG > 0.4). This suggests that the seed coating technology affected adversely the seed vitality at a more intense drought simulation. Compared with the US treatment, the DS percentage value was 30% higher at the concentration of 0.4 mol, and the difference in the number of dead seeds further grew with the increasing concentration. It is possible to deduce from these data that the use of WASP technology in the case of CS affected not only germination but also the percentage of dead and hard seeds as the share of hard seeds was at all times higher in the US treatment (by more than 5%) compared with the CS treatment (Figure 4).

The measured data indicate that the drought simulation affected the capacity of seeds to germinate because germination capacity is the most sensitive growth stage of each plant, which adversely responds to drought stress [30]. In our experiment, the drought stress was induced by PEG application; this method can be considered a standard one as it is conventionally used for seed stock testing and induction of osmotic stress [8,30,31]. The two kinds of seed stock (US and CS) exhibited the germination capacity decreasing with the increasing PEG concentration, which is confirmed also by Hellal et al. [32], who claimed that the germination capacity of seeds decreases with the increasing osmotic potential of PEG.

In the submitted study, no positive influence of coating was observed on the overall average values of seed drought resistance at higher PEG concentrations. The coating of seed with hydro-absorbents has been tested over a long time with respect to their influence on the growth of plants after sowing, i.e., their effect on the emergence of plants [33]. The coating of seed with a substance that is capable of binding water is considered a possibility for mitigating the impact of drought on the emergence of seed stock [8,33]. This was not demonstrated by the overall values in the submitted experiment, though. The overall average values could have been distorted by the different responses of respective plant species to the coating technology. This is confirmed also by Vymyslický et al. [34] or Gorim and Asch et al. [33], who pointed out the effect of varieties that may influence the response to seed coating within one plant species. On the other hand, seed coating is considered a practice intended rather to improve handling and protection of plants (against mechanical or biological damage) and to a lesser extent also to enhance germination capacity [35].

### 2.3. Comparison of Germinated, Dead and Hard Seed Percentages in US and CS Treatments in the Respective Plant Species at Drought Simulation (PEG = 0.0–0.7 mol)

Results of percentage values of GS, DS and HS with respect to average values for all model plants were described and discussed in the above sub-chapter. The average values were divided into two main groups/treatments (US and CS), the main aim being the characterization of the general influence of seed coating on seed quality parameters. The effect of seed coating within the respective plant species is described in the following sub-chapter.

Prior to the assessment of seed coating technology and its effect on the seed drought resistance in the respective plant species, the response of seeds of model plants was assessed in control conditions (Table 1). The measured values indicated (*p* < 0.05) that a significant difference existed in the selected parameters (GS, DS and HS) between US and CS within the respective plant species. However, the values showed that the individual species responded differently to the coating of seeds. While in some of them, CS had a higher number of germinated seeds (*A. vulneraria*, *T. repens*, *M. albus*), the other species (*A. vulneraria* and *O. viciifolia*) responded in the opposite way. The differences in the GS of all five species were statistically significant between US and CS. Fewer significant differences were found in DS, where they were detected only in *A. vulneraria*, *T. repens* and *M. albus*. The CS treatment showed a significantly lower percentage of DS value in *T. repens* and *M. albus*. By contrast, the lower percentage DS in US was observed in *A. vulneraria*. The fewest significant differences between US and CS were found in the NHS parameter only in *A. vluneraria* and *M. albus*. In both plant species, the highest percentage of HS was always detected in the US treatment.

Vymyslický et al. [34] arrived at similar conclusions in standard conditions (absence of drought simulation). Their research showed too that the studied plant species could be divided into two groups in terms of the number of germinated seeds: those in which the seed coating positively influenced the number of germinated seeds (*M. lupulina*, *T. repens* and *M. albus*) and those in which the seed coating slightly decreased the germination capacity (*A. vulneraria* and *O. viciifolia*). Thus, the plant species could be classified into two groups in the presented study: (a) The first group of species (*M. lupulina*, *T. repens*, *M. albus*) exhibited an increased percentage of germinated seeds in CS from 2.3% (*T. repens*) to 30.5% (*M. lupulina*). (b) The other group of species (*A. vulneraria*, *O. viciifolia*) exhibited a decreased number of germinated seeds in CS on average by 9.3%. It should be added that, compared with US, the seed coating caused an increase in the number of dead seeds, namely, in Common sainfoin (from 9.3% to 22.3%) and Kidney vetch (from 5.5% to 15.0%). Some hard seeds (6.7%) were brought to germination by the coating. This fact was most striking in *M. lupulina,* where the percentage of hard seeds amounted to 32.5% in US and only 2.8% in CS (difference of 29.7%) and in *M. albus* in which the shares were 14.0% and 1.0% (difference of 13.0%).

### 2.4. Anthyllis vulneraria

The leguminous *A. vulneraria* represents a biennial clover plant that is suitable for dry conditions. It is used in clover–grass mixtures as fodder for farm animals, as a source of pasture for pollinators and as a reclamation plant [36,37].

**Figure 6:**
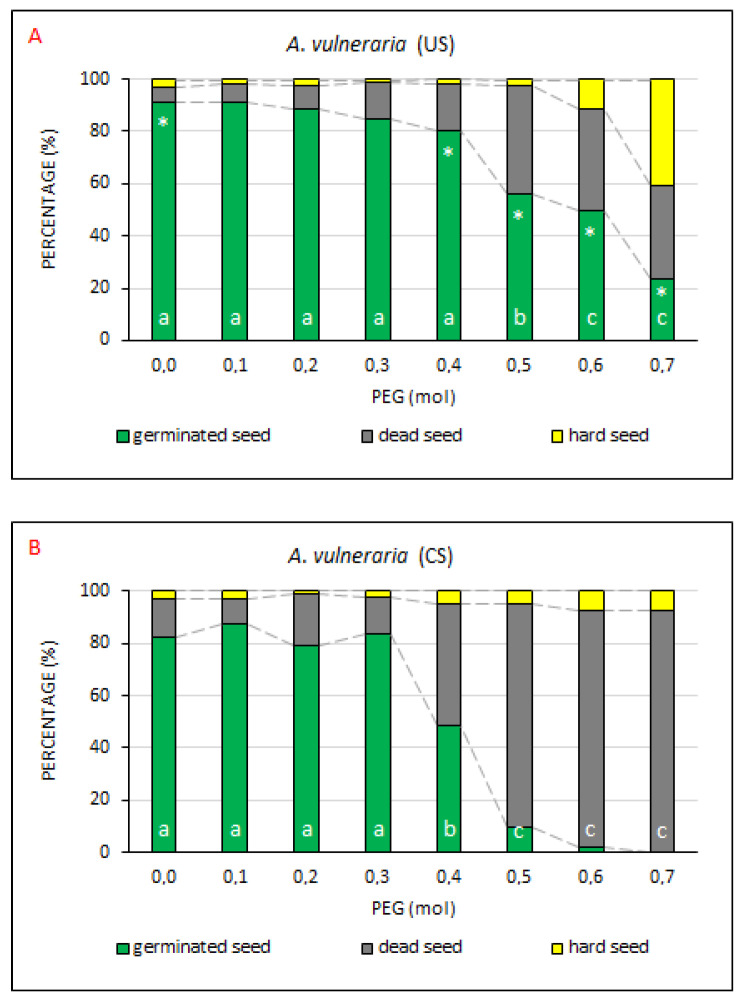
The comparisons of germinated, dead and hard seed percentages at drought simulation degrees. Average percentages of GS, DS and HS for *A. vulneraria*, the US (uncoated seeds–**A**; n = 4 ± SE) and CS (coated seeds–**B**; n = 4 ± SE) treatments at the respective PEG concentrations = 0.0–0.7 mol. Different lowercase letters indicate a significant difference (ANOVA post-hoc Tukey’s HSD test; *p* < 0.05) between the percentage of GS in the US and CS treatments. The * symbol represents a significant difference in the percentages of GS between US and CS at a specific PEG concentration.

In this respect, a higher resistance to drought simulation was expected, which was corroborated also by the measured values (Figure 6) when the percentage of GS was higher than 80% in the US treatment up to the PEG concentration of 0.4 mol, which can be considered a simulation of moderately intense drought [29]. From that concentration a significant decline in germination capacity occurred. Despite this decline, the germination capacity was higher in US as compared with CS at all PEG concentrations (Appendix B, Table A2, Table A3 and Table A4). In the control (with no PEG treatment), GS (91.3%) in the US treatment was on average 9% higher as compared with the CS treatment (82.0%), the difference being statistically significant. The percentage of dead seeds in CS (15.0%) was also statistically significantly higher than in US (5.5%). The proportion of hard seeds in US (3.3%) and CS (3.0%) treatments was essentially the same. Statistically significant differences in the representation of germinated and dead seeds between US and CS were detected in the PEG treatment ≥0.4 mol. Differences in the percentage of hard seeds between US and CS were non-significant.

The measured values show that the seeds of *A. vulneraria* did not respond positively to the used technology of coating with respect to their resistance to PEG drought simulation (Appendix C, Table A5). It is also possible to presume that the seeds exhibited a naturally increased resistance to drought stress. In contrast to *M. lupulina*, *T. repens* and *M. albus*, in which the coating of seeds slightly increased the germination capacity, the uncoated seeds of *A. vulneraria* exhibited a higher drought resistance at all PEG concentrations than the coated seeds. This might relate to the fact that ecology of germination is rather complex in *A. vulneraria*. As mentioned by Sterk et al. [37], an important role is played also by the seed size and the place of seed origin on the plant. Earlier developed seeds germinate slower than those developed later. Seeds of smaller size germinate faster than larger seeds. Earlier developed flower heads produce a somewhat higher proportion of large seeds than later developed flower heads.

### 2.5. Medicago lupulina

**Figure 7:**
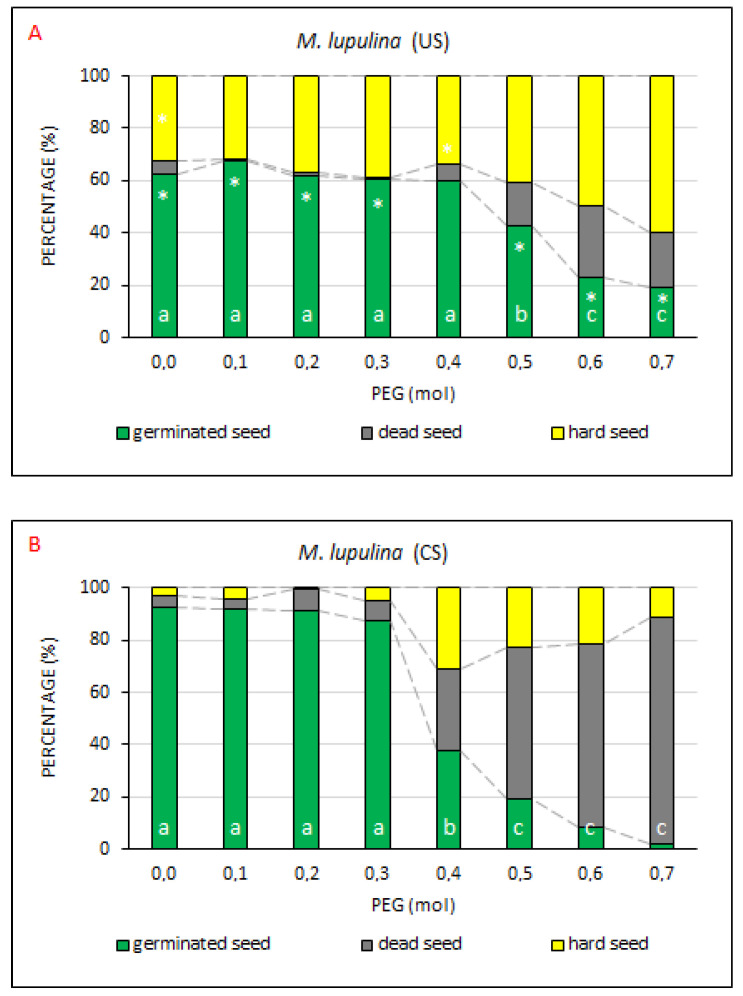
The comparison of germinated, dead and hard seed percentages at drought resistance degrees. Average percentages of GS, DS and HS for *M. lupulina*, the (**A**) US (uncoated seeds; n = 4 ± SE) and (**B**) CS (coated seeds; n = 4 ± SE) treatments at the respective PEG concentrations = 0.0–0.7 mol. Different lowercase letters indicate a significant difference (ANOVA post-hoc Tukey’s HSD test; *p* < 0.05) between the percentage of GS values in the US and CS treatments. The * symbol represents a significant difference in the percentages of GS values between US and CS at a specific PEG concentration.

The values measured in *M. lupulina* (GS, DS and HS percentage; Figure 7) exhibited a different course as compared with the same parameters monitored in the seed of *A. vulneraria*. The seed of this plant species was markedly less drought resistant (GS lower by more than 20% at the respective PEG concentrations).

The finding was rather surprising as *M. lupulina* ranks with honey crops particularly suitable for dry soils. It is both an annual and a winter crop, which is maintained in perennial stands by shedding seeds, and is used as a component in special-purpose grass mixtures on perennial and temporary meadows and pastures, useful also when undersown for green manure [38]. According to Fan et al. [39] and Amer et al. [40], *M. lupulina* has a potential to be used for phytoremediation and biomass production on soils with high concentrations of heavy metals.

It was further found out that the GS in the control sample (with no PEG treatment) of US treatment (62.3%) was on average 30.5% lower (Figure 7) as compared with the CS treatment (92.8%). The difference was statistically significant. The share of dead seeds in the US treatment (5.3%) did not differ significantly from the CS treatment (4.5%). The percentage of hard seeds was statistically significantly higher in the US treatment (32.5%) than in the CS treatment (2.8%).

In the control samples and in the samples exposed to PEG drought simulation (ranging from 0.1 to 0.4 mol; Appendix C, Table A6), the percentage of GS was considerably lower (60.3–67.5%) in the US treatment as compared with the CS treatment (87.3–92.8%). By contrast, the percentage of hard seeds was markedly higher in the US treatment as compared with the CS treatment (2.8–5.3%), ranging from 32.0% to 39.0% at PEG concentrations from 0.1 to 0.3 mol. A conspicuous decrease in the proportion of germinated seeds occurred only at the PEG concentration of 0.5 mol (42.5%), which was lower by 20% as compared with the control (62.3%). Along with the decreased level of germination, the proportion of HS increased, and to a lesser extent also the proportion of DS, the shares of GS, DS and HS being 19.0%, 21.3% and 59.8%, respectively, at the PEG concentration of 0.7 mol. By contrast to US, the number of dead seeds increased in CS with the increasing PEG concentration. The proportion of hard seeds in CS was the highest at the PEG concentration of 0.4 mol, and decreased with the further growing concentration. At the PEG concentration of 0.7 mol, the shares of GS, DS and HS were 2.3%, 86.8% and 11.0%, respectively (Appendix C, Table A6).

The measured values demonstrated that the seed coating technology has a potential to improve seed germination capacity at lower intensities of drought simulated by the application of PEG in concentrations ranging from 0.1 to 0.3 mol as compared with the absence of seed coating technology. At a higher concentration, the germination capacity sharply fell with the decline being more intense in the coated seeds. Comparing CS and US, the use of CS significantly further decreased the number of hard seeds by up to 30–40%. On the other hand, higher PEG concentrations increased the number of dead seeds in CS. The higher number of germinated seeds in CS than in US can be explained by the fact that the seeds of *M. lupulina* exhibit a non-dormant phase during their development [40,41]. During this phase, which lasts approximately 10 days in field conditions, the seeds can instantly germinate if the conditions become favorable. Phase duration depends on the environment in which the seeds ripen as well as on the genotype of mother plant. Thus, the seed coating can apparently discontinue dormancy in hard seeds. Sidhu et al. [42] claimed, however, that a majority of common methods for dormancy discontinuation have only a low effect on the seeds of *M. lupulina* (e.g., stratification, scarification with unconcentrated sulphuric acid, various concentrations of potassium nitrate and gibberellinic acid or priming mixtures of methanol and chloroform). Nevertheless, thorough scarification will bring to life practically all viable seeds. Sidhu et al. [42] also eliminated dormancy of *M. lupulina* by exposing moistened seeds to high temperatures and achieved a maximum germination capacity of 92% after 135 min of exposure to a temperature of 80 °C.

Based on results from experiments with two *Medicago* genus species, Patanè et al. [43] concluded that the share of hard seeds depends very strongly on the degree of stress caused by water deficit during the seed development and ripening. The fact apparently applies to other clover plant species, too.

Fully ripe seeds of *M. lupulina* can stay viable in the soil for many years [42]. Medvedev et al. [44] informed that 10–11 years of storage had only a low influence on viability or germination but reduced the number of hard seeds.

### 2.6. Trifolium repens

Another plant studied in detail with respect to the response of its seeds to the simulated drought stress and use of coating technology to mitigate its effect was *T. repens*. According to Marshall et al. [45], *T. repens* is one of the most widely spread and important clover plants because it can be used in agriculture in several ways: (a) cover crop; (b) animal feed; (c) restoration of soil fertility. Therefore, it is important to study the resistance of its emerging seeds in conditions of climate change when periods of drought occur more frequently and are longer [46], which adversely affects soil fertility and emergence of plants, not only in central Europe [47]. This plant species is grown in field cultures not only as a fodder plant but in a dry and green state is also suitable for grazing stands, for both ornamental lawns and technical greens as well as for bee pasture [38].

**Figure 8:**
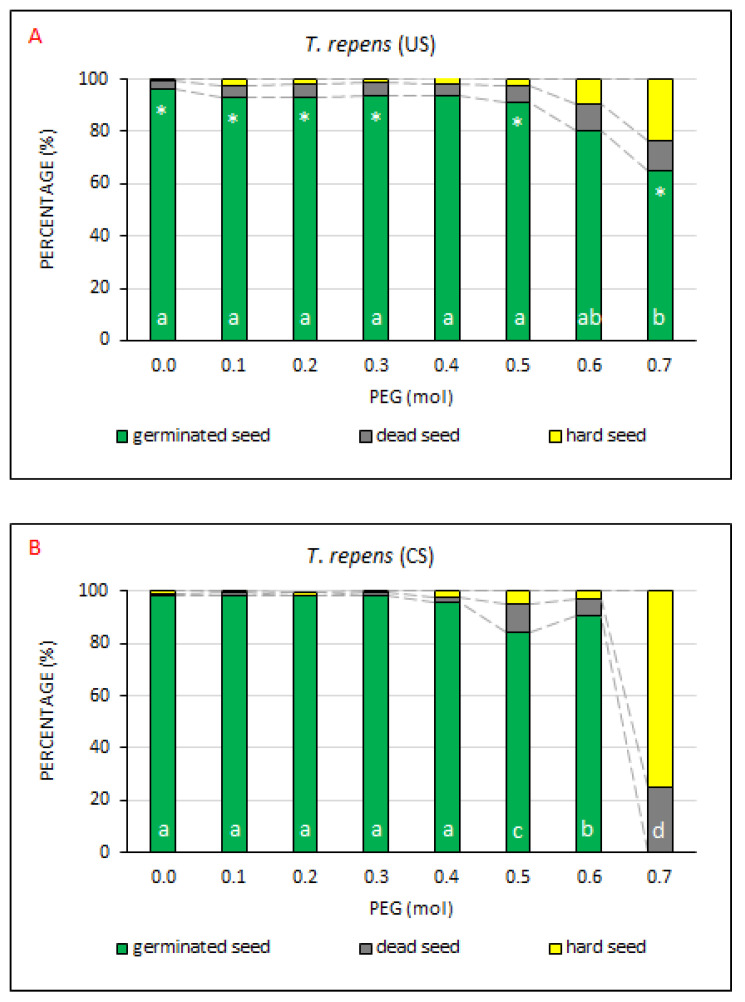
The comparison of germinated, dead and hard seed percentages at drought simulation degrees. Average percentages of GS, DS and HS for *T. repens*, the (**A**) US (uncoated seeds; n = 4 ± SE) and (**B**) CS (coated seeds; n = 4 ± SE) treatments at the respective PEG concentrations = 0.0–0.7 mol. Different lowercase letters indicate a significant difference (ANOVA post-hoc Tukey’s HSD test; *p* < 0.05) between the percentage of GS values in the US and CS treatments. The * symbol represents a significant difference in the percentages of GS values between US and CS at a specific PEG concentration.

The measured values of GS proportion from all seeds (%) in the US treatment (Figure 8A) indicated that *T. repens* exhibited a higher drought resistance, since no significant differences were found up to the PEG concentration of 0.6 mol (Appendix C, Table A7) compared to the control without the drought stress. The GS values ranged from 91% to 96.3%. A statistically significant decrease in the germinated seeds occurred only with the concentration of 0.7 mol (GS = 65.3%; DS = 11.3%; HS = 23.5%). In the CS treatment, the strong resistance to drought was observed, too, but the decline of seed germination capacity was lower (slower) as compared with the abovementioned plants. Although a significant germination decline was recorded already at a concentration of 0.5 mol PEG as compared with the control, which was a lower intensity of drought simulation than that at which a significant decline was recorded in the US treatment, the GS subsequently significantly increased as compared with the control at a PEG concentration of 0.6 mol.

As to total mean values (Appendix B, Table A2), the difference in the percentages of GS between US (88.2%) and CS (83.1%) was lower than in the other model plant species and was not statistically significant. In the case of percentage of DS or HS parameters (Appendix B, Table A3 and Table A4), no significant differences were found between the US and CS treatment, either.

Marshall et al. [45] claimed that *T. repens* is less tolerant to drought than the other perennial clover plants of temperate zone due to its shallow root system and incapacity of efficient transpiration control [48]. That was probably the reason why the seed of this plant positively responded to coating both in terms of total GS values and in terms of GS values in the simulation of drought with PEG up to the concentration of 0.6 mol. Then the germination became inhibited. According to Barbour et al. [49], too, stress due to water deficit is one of the most important obstacles to the growth and resistance of *T. repens*.

### 2.7. Melilotus albus

**Figure 9:**
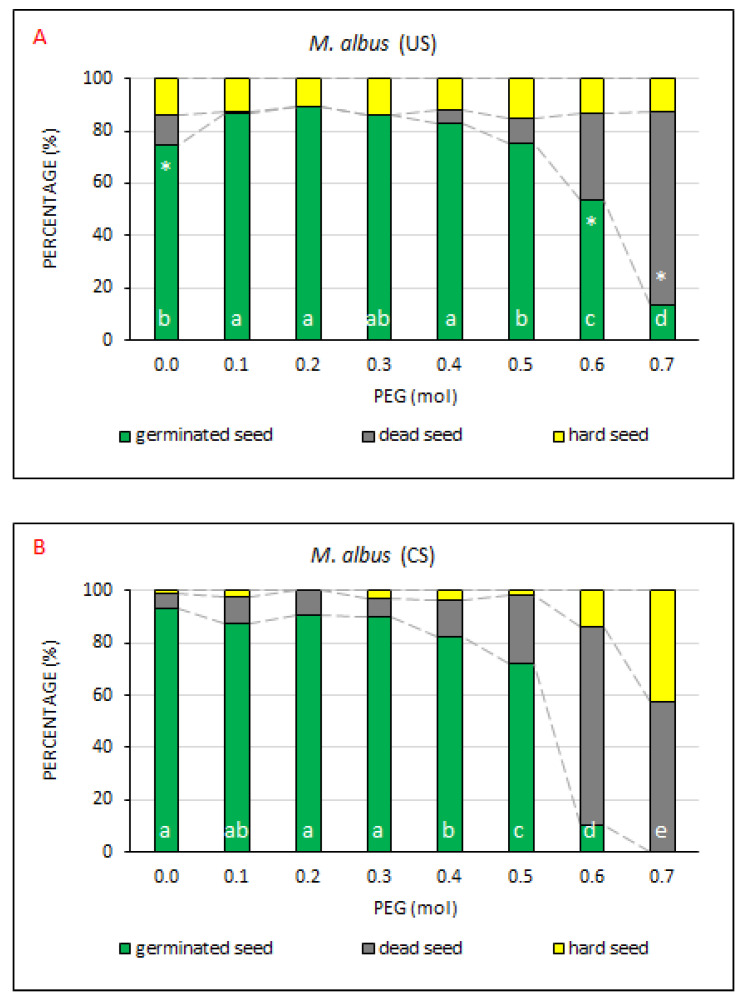
The comparison of germinated, dead and hard seed percentages at drought simulation degrees. Average percentages of GS, DS and HS for *M. albus*, the (**A**) US (uncoated seeds; n = 4 ± SE) and (**B**) CS (coated seeds; n = 4 ± SE) treatments at the respective PEG concentrations = 0.0–0.7 mol. Different lowercase letters indicate a significant difference (ANOVA post-hoc Tukey’s HSD test; *p* < 0.05) between the percentages of GS values in the US and CS treatments. The * symbol represents a significant difference in the percentages of GS values between US and CS at a specific PEG concentration.

*M. albus* is usually used as a fodder plant and reclamation plant species for infertile soils [50]. It was originally a biennial plant but some annual varieties have been bred as well. It can grow on soils poor in humus, tolerates drought, prefers sunlit sites, and abhors waterlogged and heavy soils. The plant contains a large amount of coumarin, which causes rubbed stalks, leaves and especially flowers to smell nice [38]. The measured values indicated (Figure 9) that, similar to the case of the abovementioned plant species, the PEG concentration ranging from 0.4 mol to 0.5 mol represented a limiting value, above which the seed began to respond to drought stress very negatively.

On the other hand, at a mild drought simulation (PEG 0.1–0.4 mol) the proportion of GS in US significantly increased. The percentage of GS value in the US treatment was adversely affected by drought simulation only from 0.6 mol. Thus, a statistically significant decline in the average representation of GS from the total number of tested seeds (to 53.8%) and a simultaneous increase in the average representation of DS (33.0%) occurred at the PEG concentration of 0.6 mol. The proportion of HS remained approximately constant, with no significant differences, ranging on average around 13%. The values of germinated seeds were around 90% at the PEG concentrations from 0.0 mol to 0.3 mol. At the concentration of 0.4 mol, the number of germinated seeds obviously began to decline and the number of dead seeds started to grow.

It is therefore possible to state that the given model plant species requires a “mild drought stress” to intensify the process of germination. Pros of this drought simulation were characterized, for example, by Haivan et al. [51]. On the other hand, the data indicated once again that *M. albus* belongs in the group of crops that respond positively to the process of seed coating, and the process of seed coating does not lead to its improved drought resistance. This is obvious if all three studied parameters (GS, DS, HS) are compared with respect to their total mean values (Appendix B). A significant difference was not recorded in even one of these parameters. However, as to the respective PEG concentrations, partial differences were found between the US and CS treatments. In the CS treatment, *M. albus* exhibited a higher drought resistance than in the US treatment at PEG concentrations ranging from 0.0 mol to 0.4 mol (Appendix C, Table A8). In this range, CS reached a slightly higher germination as compared with US, but with no statistical significance. The only statistically significant differences in the germination capacity were recorded in the control treatment (higher in CS) and in treatments with the PEG concentrations of 0.6 and 0.7 (higher in US). In US, non-germinating seeds were represented rather by hard seeds, while in CS non-germinating seeds were dead seeds. A pronounced decline in the number of germinated seeds occurred only at PEG concentrations of 0.5–0.7 mol and was significant as compared with the US treatment.

*M. albus* produces a variable share of hard seeds that are dormant due to water impermeability of the seed coat [52]. The share of hard seeds greatly fluctuates. According to Clark et al. [38], it can amount to 50% and more. Over 90% of seeds of *M. albus* gathered from roads and pastures near Leuven, Belgium, in July and August were hard [53]. Although the published reports have introduced very variable results it seems that hard seeds can survive in the soil for a time period estimated up to 81 years [54]. Factors affecting the amount of hard seeds have not been described in available literature sources [52]. On the other hand, in the presented experiment the proportion of hard seeds ranged only from 10.8 to 15.3% in US treatment and from 0.0 to 42.3% in CS treatment.

The difference between data declared by the above authors and this experiment can be explained based on the study publicized by Paiaro et al. [55], who found out that the germination capacity of *M. albus* was significantly lower (*p* = 0.0069) in field conditions (6.7%) than in laboratory conditions. Thus, results of greenhouse experiments may not be fully feasible in field conditions.

### 2.8. Onobrychis viciifolia

Another tested seed was that of *O. viciifolia,* which can be considered one of the most valuable fodder plants cultivated in Europe since the 16th century. The fodder for which *O. viciifolia* is grown is of high quality, its yields are high, it is readily digested and its nutritional value is high, too. It was demonstrated that condensed tannins present in Common sainfoin give the plant antihelmintic properties, improve the utilization of proteins and prevent flatulence; they can also potentially reduce greenhouse gas emissions [56]. Thanks to its extensive root system, reaching a depth of up to 1 m and width of 2 m, Common sainfoin is very resistant to drought [57].

**Figure 10:**
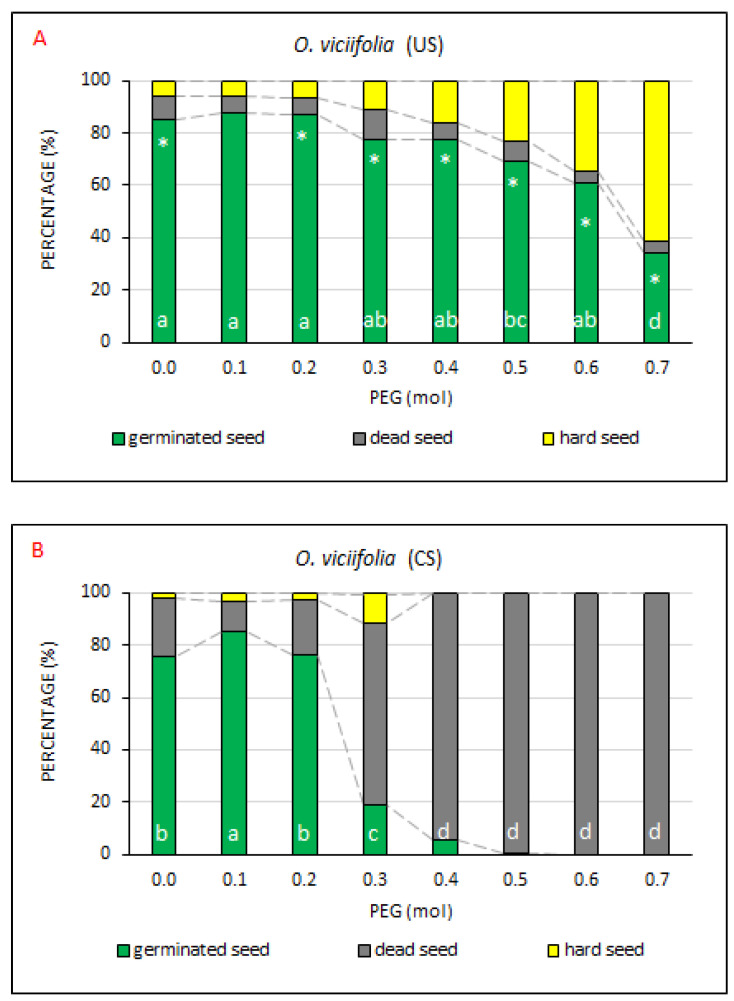
The comparison of germinated, dead and hard seed percentages at drought simulation degrees. Average percentages of GS, DS and HS for *O. viciifolia*, the (**A**) US (uncoated seeds; n = 4 ± SE) and (**B**) CS (coated seeds; n = 4 ± SE) treatments at the respective PEG concentrations = 0.0–0.7 mol. Different lowercase letters indicate a significant difference (ANOVA post-hoc Tukey’s HSD test; *p* < 0.05) between the percentages of GS values in the US and CS treatments. The * symbol represents a significant difference in the percentages of GS values between US and CS at a specific PEG concentration.

The values measured for the respective PEG concentrations (Figure 10) and total means (Appendix C, Table A9) indicated that the seed of *O*. *viciifolia* did not respond positively to the coating. Significant differences were found in all parameters (GS, DS and HS–Appendix C, Table A9), which confirmed a higher germination capacity and a lower content of DS in the US treatment.

As mentioned above, the US treatment exhibited better seed vitality. At the PEG concentrations of 0.0–0.4 mol, the percentages of GS ranged from 77.8 to 87.5%. A statistically significant decline was recorded at the PEG concentrations of 0.5–0.7 mol. The value of 0.5 mol (i.e., 15 wt %) PEG can be considered a limiting concentration, above which an intense drought affects the seed of model plants (Wu et al., 2019). The decreasing germination was accompanied by the increasing proportion of HS. The DS percentage remained more or less constant within an interval of 4.3–11.0%. The percentage of GS value at the PEG concentration of 0.7 was 34.0%; DS and HS amounted to 4.3% and 61.8%, respectively.

Compared to that, GS and DS percentages in the CS treatment reached on average 79% and 18%, respectively, at the PEG concentrations of 0.0–0.2 mol. At the concentration of 0.3 mol, the proportion of GS sharply (*p* < 0.05) declined to 19.0%, with the numbers of dead seeds and hard seeds increasing to 69.3 % and 11.5%, respectively. At the PEG concentrations 0.4–0.7 mol the number of germinated seeds, as well as the number of hard seeds, neared or were equal to 0.0%, while the number of dead seeds neared or was equal to 100%.

In *O. viciifolia*, the US treatment exhibited a greater drought resistance (*p* < 0.05) than the CS treatment at the PEG concentrations > 0.1 mol (Figure 10, Table 2). The values of germination capacity corresponded to those detected in the laboratory test of germination capacity conducted by Küchenmeister et al. [58] with the uncoated seed stock and without the application of PEG (mean 89 ± 7% SE).

Compared with the US treatment, CS exhibited a slightly lower proportion of germinated seeds at PEG concentrations of 0.0–0.2 mol. Moreover, at the PEG concentration of 0.3 mol, the CS treatment showed a sharp, significant decline of germination capacity and a great increase in the DS percentage. Hard seeds were totally missing at PEG concentrations ≥0.4 mol. In US, non-germinating seeds were represented rather by hard seeds, while in CS it was mainly dead seeds. Similar to *A. vulneraria*, seed coating rather suppressed the germination capacity in *O. viciifolia* [34]. A conspicuous decline in the number of germinated seeds occurred in US only at PEG concentrations of 0.5–0.7 mol.

## 3. Materials and Methods

Drought resistance of seeds of the following five fodder clover (forage legume) species was studied in the experiment: *Anthyllis vulneraria* L. (Kidney vetch), *Medicago lupulina* L. (Black medick), *Trifolium repens* L. (White clover), *Melilotus albus* Medik. (White sweet clover) and *Onobrychis viciifolia* Scop. (Common sainfoin). Seeds of the abovementioned plant species were purchased from authorized breeders:

- *A. vulneraria*—Research Institute for Fodder Crops, Ltd., Czech Republic
- *M. lupulina*—Research Institute for Fodder Crops, Ltd., Czech Republic
- *T. repens*—AGROGEN, Ltd., Czech Republic
- *M. albus*—Research Institute for Fodder Crops, Ltd., Czech Republic
- *O. viciifolia*—AGROGEN, Ltd., Czech Republic

All seeds used have been registered by the Central Institute for Supervising and Testing in Agriculture of the Czech Republic in line with EU directives for placing the seed on the EU market.

### 3.1. Seed Coating

The purchased seed was additionally cleaned so that no batch would contain undesirable admixtures. Thus, the seed was prepared for the process of coating. No chemical treatment was applied prior to the coating. The technology used for seed coating was WASP, which allows to increase the percent of seed emergence in extensive conditions with no possibility of irrigation in the critical period of seed germination, based on the principle of special pelletization of seeds. In addition to the coat consisting of a combination of fertilizers for the initial germination of seeds (humic acids for faster germination, bio-stimulating agents for plant health and root activators), it also contained an absorption component (Hydrogel), which is a water reservoir, thus forming the fundamental seed coat component [5].

### 3.2. Drought Stress Simulation

Drought stress was simulated by applying the solution of polyethylenglycol (PEG 8000) in seven different concentrations (from 0.1 mol to 0.7 mol). Treatments of the experiment were represented by seed stocks of five different indicator plants, which were exposed to seven degrees of different osmotic pressure induced by different PEG solution concentrations (at all times by 0.1 mol). Control was distilled water, i.e., PEG = 0 mol. Each of the experimental treatments, i.e., PEG concentration, had four repetitions in the seed stock of each model plant. An overview of experimental treatments is presented in Table 3. The action of PEG and distilled water on the seed stock of model plants was implemented according to Muscolo et al. [8]: the seeds were chosen to have a homogeneous size, then they were placed into Petri dishes of 8 cm in diameter with either 3 cm^3^ of distilled water (control with no stress) or PEG in a concentration from 0.1 mol to 0.7 mol (simulation of drought). The PEG solution (Sigma-Aldrich, member of Merck KGaA, Darmstadt, DE) used was of density 1.08 g/cm^3^.

### 3.3. Assessment of Germination

Germination was assessed based on the methodology of the International Seed Testing Association [59], according to which the seed germination percent is determined by testing 100 seeds in four repetitions, i.e., 400 seeds in each treatment. According to the methodology, a process of precooling was applied, first, in order to discontinue dormancy. Seeds were spread on the moist filter paper placed on Petri dishes 8 cm in diameter. The Petri dishes prepared in this way were filled with either distilled H_2_O or PEG (see the above sub-chapter) and placed in the thermostat (LOVIBOND TC 255 S, Tintometer Limited, Amesbury, UK) at a temperature of 5 °C for 4 days. After the end of the precooling process, the Petri dishes were kept at a temperature of 20 °C. Germinated seeds were counted every 4 days (Day 5–Day 22). Germination capacity of the individual treatments was determined after the end of all repetitions.

Germination capacity of seeds was calculated according to the following formula [60]:

(1) $$Germinationpercentage\left(\%\right)=\frac{Totalnumberofgerminatedseeds}{Totalseedplacedforgermination}\times 100.$$

Apart from the percentage of germinated seeds (GS), dead seeds (DS) and hard seeds NHS) were counted, too. An overview of determined parameters is presented in Table 4 and Table 5.

### 3.4. Statistic Data Processing

The measured data were analyzed and processed using the Statistica 12 (Dell Software, Round Rock, TX, USA) program. First, an input analysis of data was made to establish their homogeneity and aptness for further analyses. Then variances of individual values were calculated, which were analyzed using one-factor ANOVA in combination with the post-hoc Tukey HSD test and the pair t-test, which were used to identify the significance of differences in selected parameters. All analyses were made at a significance level *p* < 0.05.

## 4. Conclusions

Based on drought stress simulation through the application of various PEG concentrations on the seeds of tested plant species, it is possible to judge that with respect to seed germination, seed coating can be expedient only in conditions of mild drought. Mild drought was simulated by the application of PEG at concentrations up to 0.3 mol. At higher concentrations, the germination capacity of coated seeds significantly declined as compared with that of uncoated seeds. This is confirmed by the values of germination capacity presented in Table 2, which indicate that despite differences among the respective plant species, the germination capacity at all times decreased (in all treatments) with the increasing PEG concentrations. PEG concentrations of 0.3–0.4 mol can be considered breaking as a sharp decline in germination capacity always occurred after this limit was crossed, with the coated seeds exhibiting in a majority of cases a significantly greater decline of germination capacity than the uncoated seeds.

Important factors affecting the seed germination capacity are dormancy, its type, intensity and time course. Seed coating can discontinue dormancy in hard seeds in certain cases. The fact showed most in *M. lupulina,* in which the coating discontinued dormancy in the form of hard seeds, thus increasing the germination capacity by up to 30% (Appendix C, Table A6). In this respect, the seed coating was somewhat less efficient in *M. albus* and *O. viciifolia,* in which it resulted in the death of some hard seeds. At higher PEG concentrations, the share of dead seeds in CS increased as compared with US, in which it was rather the number of hard seeds that increased at higher PEG concentrations.

Coated seed can be positively used in *M. lupulina*, *M. albus* and *T. repens*. In these plant species, the coating of seeds increased the germination capacity.

Uncoated seed can be used in *A. vulneraria* and *O. viciifolia*. In these plant species, the coating of seeds decreased the germination capacity.

Laboratory tests of germination capacity using PEG represent a fast and effective possibility for testing the seed of various plant species and their varieties with respect to their capability of resistance to stress induced by water deficit.

## Figures and Tables

**Table 1:** Percentages of germinated, dead and hard seeds in the US and CS treatments with the distinction of individual model plants and absence of drought simulation.

Species	Variant (US/CS)	PEG Concentration (mol)	Germination Capacity		
			GS (%)	DS (%)	HS (%)
*A. vulneraria*	US	0.0	91.3 ± 2.09 a	5.5 ± 1.04 b	3.3 ± 1.49 a
	CS		82.0 ± 1.73 b	15.0 ± 0.91 a	3.0 ± 1.08 a
*M. lupulina*	US	0.0	62.3 ± 2.28 b	5.3 ± 1.32 a	32.5 ± 2.10 a
	CS		92.8 ± 1.70 a	4.5 ± 1.32 a	2.8 ± 1.11 b
*T. repens*	US	0.0	96.3 ± 0.63 b	3.3 ± 0.75 a	0.5 ± 0.49 a
	CS		98.5 ± 0.65 a	0.5 ± 0.28 b	1.0 ± 0.70 a
*M. albus*	US	0.0	74.5 ± 2.78 b	11.5 ± 1.29 a	14.0 ± 1.77 a
	CS		93.5 ± 0.64 a	5.5 ± 0.66 b	1.0 ± 0.41 b
*O. viciifolia*	US	0.0	85.0 ± 2.74 a	9.3 ± 3.56 b	5.8 ± 2.78 a
	CS		75.8 ± 2.56 b	22.3 ± 2.56 a	2.0 ± 0.0 a
Mean	US	0.0	81.9 b	7.0 a	11.2 a
Mean	CS		88.5 a	9.6 b	2.00 b
Difference (US vs. CS)			6.7	2.6	−9.3

Average percentage values of GS (germinated seeds), DS (dead seeds) and HS (hard seeds) for the US (uncoated seeds; n = 4 ± SE) and CS (coated seeds; n = 4 ± SE) treatments within the respective plant species. Different lowercase letters indicate a significant difference (ANOVA post-hoc Tukey’s HSD test; *p* < 0.05) in the individual plant species between the US and CS groups.

**Table 2:** Percentages of germinated seeds in the US and CS treatments with the distinction of individual model plant species and drought simulation degrees.

	Uncoated Seeds									Coated Seeds								
Species	PEG Concentration (mol)																	
	**0.0**	**0.1**	**0.2**	**0.3**	**0.4**	**0.5**	**0.6**	**0.7**	**Mean**	**0.0**	**0.1**	**0.2**	**0.3**	**0.4**	**0.5**	**0.6**	**0.7**	**Mean**
*A. vulneraria*	91.3	91.0	88.3	84.5	80.3	55.8	49.5	23.3	70.5	82.0	87.3	79.0	83.5	48.3	9.8	2.0	0.0	49.0
*M. lupulina*	62.3	67.5	61.5	60.3	60.0	42.5	23.0	19.0	49.5	92.8	91.8	91.5	87.3	37.8	19.5	8.3	2.3	53.9
*T. repens*	96.3	92.8	93.0	93.5	93.8	91.0	80.0	65.3	88.2	98.5	98.3	98.3	98.5	95.5	84.5	90.8	0.0	83.0
*M. albus*	74.5	87.0	89.0	86.0	83.0	75.5	53.8	13.3	70.3	93.5	87.8	90.8	90.3	82.3	72.3	10.3	0.0	65.9
*O. viciifolia*	85.0	87.5	87.0	77.8	77.8	69.0	60.8	34.0	72.3	75.8	85.0	76.5	19.0	5.3	0.3	0.0	0.0	32.7
Mean	81.9	85.2	83.8	80.4	79.0	66.8	53.4	31.0	70.2	88.5	90.0	87.2	75.7	53.8	37.3	22.3	0.5	56.9
Germination range (%)																		
80.0–100.0																		
60.0–79.9																		
40.0–59.9																		
20.0–49.9																		
0.0–19.9																		

Average values of percentages of GS (germinated seeds) for US (uncoated seeds; n = 4 ± SE) and CS (coated seeds; n = 4 ± SE) treatments (different concentration of PEG) within the respective plant species. Values marked in red indicate a significant difference (ANOVA post-hoc Tukey’s HSD test; *p* < 0.05) between the US and CS treatments in the individual plant species at a specific PEG concentration.

**Table 3:** Overview of laboratory experiment.

	Treatment–Simulation of Drought → Concentration of PEG (mol)	
Indicator Plant	Uncoated (US)	Coated (CS)
*A. vulneraria*	0.0; 0.1; 0.2; 0.3; 0.4; 0.5; 0.6; 0.7	0.0; 0.1; 0.2; 0.3; 0.4; 0.5; 0.6; 0.7
*M. lupulina*	0.0; 0.1; 0.2; 0.3; 0.4; 0.5; 0.6; 0.7	0.0; 0.1; 0.2; 0.3; 0.4; 0.5; 0.6; 0.7
*T. repens*	0.0; 0.1; 0.2; 0.3; 0.4; 0.5; 0.6; 0.7	0.0; 0.1; 0.2; 0.3; 0.4; 0.5; 0.6; 0.7
*M. albus*	0.0; 0.1; 0.2; 0.3; 0.4; 0.5; 0.6; 0.7	0.0; 0.1; 0.2; 0.3; 0.4; 0.5; 0.6; 0.7
*O. viciifolia*	0.0; 0.1; 0.2; 0.3; 0.4; 0.5; 0.6; 0.7	0.0; 0.1; 0.2; 0.3; 0.4; 0.5; 0.6; 0.7

**Table 4:** Overview of assessed model plant species.

Indicator Plant	
Plant Species	Common Name
*Anthyllis vulneraria* L.	Kidney vetch
*Medicago lupulina* L.	Black medick
*Trifolium repens* L.	White clover
*Melilotus albus* Medik.	White sweet clover
*Onobrychis viciifolia* Scop.	Sainfoin

**Table 5:** Overview of determined parameters and abbreviations used.

Measured Parameter	Abbreviation	Seeds	Abbreviation
Percentage of germinated seeds	GS	Uncoated	US
Percentage of dead seeds	DS	Coated	CS
Percentage of hard seeds	HS		

## Data Availability

Not applicable.

## Appendix A. Effect of Seed Coating on Germination

**Table A1:** Results of t-test for dependent samples.

Variable	Average	N	Difference	t	Significance (P)	Confidence Interval (−95%)	Confidence Interval (−95%)
GS–US	70.1	160	−13.25	−5.9461	0.000000017	−17.65	−8.84
GS–CS	56.9	160					

Comparison of overall average of percentages of germinated seeds in the CS (n = 160) and US (n = 160) treatments without distinction of plant species at drought simulation (PEG = 0.0–0.7 mol). T-test results for dependent samples (*p* < 0.05) are shown. US = Uncoated Seeds, CS = Coated Seeds.

## Appendix B. Results of Tukey’s HSD Test—General Overview of Drought Simulation on Germination

**Table A2:** Results of Tukey’s HSD test—GS, all plants.

Plant	PEG Concentration	US–NGS	CS–NGS	Difference	Tukey’s HSD (*p* Value)
*A. vulneraria*	0.0–0.7	70.5	49.0	21.5	0.0105
*M. lupulina*	0.0–0.7	49.5	53.9	-4.4	0.5772
*T. repens*	0.0–0.7	88.2	83.1	5.1	0.4040
*M. albus*	0.0–0.7	70.3	65.9	4.4	0.5761
*O. viciifolia*	0.0–0.7	72.3	32.7	39.6	0.0001

Comparison of average percentages of germinated seeds (GS) in the CS (n = 32) and US (n = 32) treatments at drought simulation (PEG = 0.0–0.7 mol) for the respective plants. Values marked in red indicate significant differences (*p* < 0.05). US = Uncoated Seeds, CS = Coated Seeds.

**Table A3:** Results of Tukey’s HSD test—DS, all plants.

Plant	PEG Concentration	US–DS	CS–DS	Difference	Tukey’s HSD (*p* Value)
*A. vulneraria*	0.0–0.7	21.3	46.6	−25.4	0.0007
*M. lupulina*	0.0–0.7	10.0	33.8	−23.8	0.0003
*T. repens*	0.0–0.7	6.3	5.8	0.4	0.7853
*M. albus*	0.0–0.7	16.7	25.7	−9.0	0.1565
*O. viciifolia*	0.0-0.7	7.0	64.9	−57.9	0.0001

Comparison of average percentages of dead seeds (DS) in the CS (n = 32) and US (n = 32) treatments at drought simulation (PEG = 0.0–0.7 mol) for the respective plants. Values marked in red indicate significant differences (*p* < 0.05). US = Uncoated Seeds, CS = Coated Seeds.

**Table A4:** Results of Tukey’s HSD test—HS, all plants.

Plant	PEG Concentration	US–HS	CS–HS	Difference	Tukey’s HSD (*p* Value)
*A. vulneraria*	0.0–0.7	8.3	4.3	4.0	0.1495
*M. lupulina*	0.0–0.7	40.5	12.4	28.2	0.0001
*T. repens*	0.0–0.7	5.5	11.1	-5.6	0.2455
*M. albus*	0.0–0.7	13.0	8.4	4.6	0.0674
*O. viciifolia*	0.0-0.7	20.7	2.4	18.3	0.0001

Comparison of average percentages of hard seeds (HS) in the CS (n = 32) and US (n = 32) treatments at drought simulation (PEG = 0.0–0.7 mol) for the respective plants. Values marked in red indicate significant differences (*p* < 0.05). US = Uncoated Seeds, CS = Coated Seeds.

## Appendix C. Results of Tukey’s HSD Test—Drought Simulation Degrees and Their Effects on Germination

**Table A5:** Results of Tukey’s HSD test—*A. vulneraria*.

Plant	PEG Concentration	US–GS	CS–GS	Tukey’s HSD (*p* Value)	US–DS	CS–DS	Tukey’s HSD (*p* Value)	US–HS	CS–HS	Tukey’s HSD (*p* Value)
*A. vulneraria*	0.0	91.3	82.0	0.0147	5.5	15.0	0.0007	3.3	3.0	0.8967
*A. vulneraria*	0.1	91.0	87.3	0.1241	7.0	9.8	0.4784	2.0	2.0	1.0000
*A. vulneraria*	0.2	88.3	79.0	0.0716	9.0	19.8	0.0028	2.8	1.3	0.6178
*A. vulneraria*	0.3	84.5	83.5	0.6756	14.3	14.0	0.9204	1.3	2.5	0.3677
*A. vulneraria*	0.4	80.3	48.3	0.0049	17.8	46.8	0.0069	2.3	5.0	0.1574
*A. vulneraria*	0.5	55.8	9.8	0.0002	41.8	85.3	0.0002	2.5	5.0	0.1341
*A. vulneraria*	0.6	49.5	2.0	0.0002	38.8	90.3	0.0002	11.8	7.8	0.1935
*A. vulneraria*	0.7	23.3	0.0	0.0540	36.0	92.3	0.0003	40.8	7.8	0.0209

Comparison of average percentages of germinated seeds (GS), of dead seeds (DS) and hard seeds (HS) in the CS (n = 4) and US (n = 4) treatments at drought simulation (PEG = 0.0–0.7 mol) for *A. vulneraria*. Values marked in red indicate significant differences (*p* < 0.05). US = Uncoated Seeds, CS = Coated Seeds.

**Table A6:** Results of Tukey’s HSD test—*M. lupulina*.

Plant	PEG Concentration	US–GS	CS–GS	Tukey’s HSD (*p* Value)	US–DS	CS–DS	Tukey’s HSD (*p* Value)	US–HS	CS–HS	Tukey’s HSD (*p* Value)
*M. lupulina*	0.0	62.3	92.8	0.0003	5.3	4.5	0.7017	32.5	2.8	0.0002
*M. lupulina*	0.1	67.5	91.8	0.0003	0.5	4.0	0.0018	32.0	4.3	0.0003
*M. lupulina*	0.2	61.5	91.5	0.0003	1.5	8.0	0.0010	37.0	0.5	0.0003
*M. lupulina*	0.3	60.3	87.3	0.0003	0.8	7.5	0.0021	39.0	5.3	0.0003
*M. lupulina*	0.4	60.0	37.8	0.1478	6.5	31.5	0.0009	33.5	30.8	0.8676
*M. lupulina*	0.5	42.5	19.5	0.0181	16.5	57.5	0.0003	41.0	23.0	0.0246
*M. lupulina*	0.6	23.0	8.3	0.0199	27.5	70.3	0.0003	49.5	21.5	0.0051
*M. lupulina*	0.7	19.0	2.3	0.0063	21.3	86.8	0.0002	59.8	11.0	0.0004

Comparison of average percentages of germinated seeds (GS), dead seeds (DS) and of hard seeds (S) in the CS (n = 4) and US (n = 4) treatments at drought simulation (PEG = 0.0–0.7 mol) for *M. lupulina*. Values marked in red indicate significant differences (*p* < 0.05). US = Uncoated Seeds, CS = Coated Seeds.

**Table A7:** Results of Tukey’s HSD test—*T. repens*.

Plant	PEG Concentration	US–GS	CS–GS	Tukey’s HSD (*p* Value)	US–DS	CS–DS	Tukey’s HSD (*p* Value)	US–HS	CS–HS	Tukey’s HSD (*p* Value)
*T. repens*	0.0	96.3	98.5	0.0470	3.3	0.5	0.0143	0.5	1.0	0.5849
*T. repens*	0.1	92.8	98.3	0.0065	5.0	1.3	0.0460	2.3	0.5	0.0205
*T. repens*	0.2	93.0	98.5	0.0009	5.3	0.3	0.0005	1.8	1.3	0.5506
*T. repens*	0.3	93.5	98.5	0.0224	5.0	1.0	0.0136	15	0.5	0.2072
*T. repens*	0.4	93.8	95.5	0.0725	3.8	2.3	0.2352	2.5	2.3	0.8346
*T. repens*	0.5	91.0	84.5	0.0199	6.3	10.5	0.0402	2.8	5.0	0.1965
*T. repens*	0.6	80.0	90.8	0.0192	10.5	6.0	0.1140	9.5	3.3	0.1232
*T. repens*	0.7	65.3	0.0	0.0009	11.3	25.0	0.0023	23.5	75.0	0.0050

Comparison of average percentages of germinated seeds (GS), dead seeds (DS) and hard seeds (HS) in the CS (n = 4) and US (n = 4) variants at drought simulation (PEG = 0.0–0.7 mol) for *T. repens*. Values marked in red indicate significant differences (*p* < 0.05). US = Uncoated Seeds, CS = Coated Seeds.

**Table A8:** Results of Tukey’s HSD test—*M. albus*.

Plant	PEG Concentration	US–GS	CS–GS	Tukey’s HSD (*p* Value)	US–DS	CS–DS	Tukey’s HSD (*p* Value)	US–HS	CS–HS	Tukey’s HSD (*p* Value)
*M. albus*	0.0	74.5	93.5	0.0008	11.5	5.5	0.0056	14.0	1.0	0.0006
*M. albus*	0.1	87.0	87.8	0.7228	0.3	9.8	0.0015	12.8	2.5	0.0003
*M. albus*	0.2	89.0	90.8	0.1657	0.3	9.3	0.0002	10.8	0.0	0,0003
*M. albus*	0.3	86.0	90.3	0.1308	0.3	6.8	0.0006	13.8	3.0	0.0018
*M. albus*	0.4	83.0	82.3	0.6335	5.3	14.3	0.0038	11.8	3.5	0.0021
*M. albus*	0.5	75.5	72.3	0.4965	9.3	26.3	0.0048	15.3	1.5	0.0002
*M. albus*	0.6	53.8	10.3	0.0004	33.0	76.3	0.0003	13.3	13.5	0.8979
*M. albus*	0.7	13.3	0.0	0.0003	74.0	57.8	0.0032	12.8	42.3	0.0003

Comparison of average percentages of germinated seeds (GS), dead seeds (DS) and hard seeds (HS) in the CS (n = 4) and US (n = 4) variants at drought simulation (PEG = 0.0–0.7 mol) for *M. albus*. Values marked in red indicate significant differences (*p* < 0.05). US = Uncoated Seeds, CS = Coated Seeds.

**Table A9:** Results of Tukey’s HSD test—*O. viciifolia*.

Plant	PEG Concentration	US–GS	CS–GS	Tukey’s HSD (*p* Value)	US–DS	CS–DS	Tukey’s HSD (*p* Value)	US–HS	CS–HS	Tukey’s HSD (*p* Value)
*O. viciifolia*	0.0	85.0	75.8	0.0489	9.3	22.3	0.0255	5.8	2.0	0.2262
*O. viciifolia*	0.1	87.5	85.0	0.3310	6.5	12.0	0.0590	6.0	3.0	0.2726
*O. viciifolia*	0.2	87.0	76.5	0.0074	6.3	21.0	0.0005	6.8	2.5	0.0275
*O. viciifolia*	0.3	77.8	19.0	0.0002	11.0	69.5	0.0002	11.3	11.5	0.9572
*O. viciifolia*	0.4	77.8	5.3	0.0002	6.3	94.8	0.0002	16.0	0.0	0.0003
*O. viciifolia*	0.5	69.0	0.3	0.0002	7.5	99.8	0.0002	23.5	0.0	0.0002
*O. viciifolia*	0.6	60.8	0.0	0.0002	4.8	100.0	0.0002	34.5	0.0	0.0002
*O. viciifolia*	0.7	34.0	0.0	0.0008	4.3	100.0	0.0002	61.8	0.0	0.0002

Comparison of average percentages of germinated seeds (GS), dead seeds (DS) and hard seeds (NHS) in the CS (n = 4) and US (n = 4) treatments at drought simulation (PEG = 0.0–0.7 mol) for *O. viciifolia*. Values marked in red indicate significant differences (*p* < 0.05). US = Uncoated Seeds, CS = Coated Seeds.
